# Contamination Levels and Phenotypic and Genomic Characterization of Antimicrobial Resistance in *Escherichia coli* Isolated from Fresh Salad Vegetables in the United Arab Emirates

**DOI:** 10.3390/tropicalmed8060294

**Published:** 2023-05-27

**Authors:** Ihab Habib, Rami H. Al-Rifai, Mohamed-Yousif Ibrahim Mohamed, Akela Ghazawi, Afra Abdalla, Glindya Lakshmi, Neveen Agamy, Mushtaq Khan

**Affiliations:** 1 Veterinary Public Health Research Laboratory, Department of Veterinary Medicine, College of Agriculture and Veterinary Medicine, United Arab Emirates University, Al Ain P.O. Box 1555, United Arab Emirates; mohamed-yousif-i@uaeu.ac.ae (M.-Y.I.M.); afra.abdalla@uaeu.ac.ae (A.A.); glindya_l@uaeu.ac.ae (G.L.); 2 High Institute of Public Health, Alexandria University, Alexandria P.O. Box 21511, Egypt; nevagamy@gmail.com; 3 Institute of Public Health, College of Medicine and Health Sciences, United Arab Emirates University, Al Ain P.O. Box 1555, United Arab Emirates; rrifai@uaeu.ac.ae; 4 Zayed Center for Health Sciences, United Arab Emirates University, Al Ain P.O. Box 15551, United Arab Emirates; mushtaq.khan@uaeu.ac.ae; 5 Department of Medical Microbiology and Immunology, College of Medicine and Health Sciences, United Arab Emirates University, Al Ain P.O. Box 1555, United Arab Emirates; akelag@uaeu.ac.ae

**Keywords:** fresh produce, *E. coli*, UAE, antibiotic resistance, WGS

## Abstract

Contaminated fresh produce has been identified as a vehicle for human foodborne illness. The present study investigated the counts, antimicrobial resistance profile, and genome-based characterization of *Escherichia coli* in 11 different types of fresh salad vegetable products (*n* = 400) sampled from retailers in Abu Dhabi and Dubai in the United Arab Emirates. *E. coli* was detected in 30% of the tested fresh salad vegetable items, with 26.5% of the samples having an unsatisfactory level (≥100 CFU/g) of *E. coli,* notably arugula and spinach. The study also assessed the effect of the variability in sample conditions on *E. coli* counts and found, based on negative binominal regression analysis, that samples from local produce had a significantly higher (*p*-value < 0.001) *E. coli* count than imported samples. The analysis also indicated that fresh salad vegetables from the soil-less farming system (e.g., hydroponic and aeroponic) had significantly (*p*-value < 0.001) fewer *E. coli* than those from traditional produce farming. The study also examined the antimicrobial resistance in *E. coli* (*n* = 145) recovered from fresh salad vegetables and found that isolates exhibited the highest phenotypic resistance toward ampicillin (20.68%), tetracycline (20%), and trimethoprim-sulfamethoxazole (10.35%). A total of 20 (13.79%) of the 145 *E. coli* isolates exhibited a multidrug-resistant phenotype, all from locally sourced leafy salad vegetables. The study further characterized 18 of the 20 multidrug-resistant *E. coli* isolates using whole-genome sequencing and found that the isolates had varying numbers of virulence-related genes, ranging from 8 to 25 per isolate. The frequently observed genes likely involved in extra-intestinal infection were *CsgA*, *FimH*, *iss*, and *afaA*. The β-lactamases gene *bla*_CTX-M-15_ was prevalent in 50% (9/18) of the *E. coli* isolates identified from leafy salad vegetable samples. The study highlights the potential risk of foodborne illness and the likely spread of antimicrobial resistance and resistance genes associated with consuming leafy salad vegetables and emphasizes the importance of proper food safety practices, including proper storage and handling of fresh produce.

## 1. Introduction

Fresh fruits and vegetables are encouraged as a healthy dietary component by both international and regional health authorities due to their significant nutritional benefits [1]. Nevertheless, the consumption of raw fresh leafy and non-leafy salad vegetable products has been associated with numerous foodborne outbreaks in different countries in recent years [2]. Bacterial pathogens and commensal organisms could contaminate fresh salad vegetable products throughout production, transportation, and handling from the field to retail. Studies have reported the occurrence of antimicrobial resistant (AMR) bacteria and resistance genes in fresh vegetable produce. Additionally, opportunistic bacteria present in fresh produce has been shown to cause infections, especially in vulnerable groups in our societies [3,4].

The most frequently reported bacteria on vegetables, fruits, and ready-to-eat salad vegetables is *Escherichia coli* (*E. coli*) [2]. Although most *E. coli* strains are commensal, some carry virulence factors that render some strains pathogenic to humans, causing intestinal and even extra-intestinal illnesses [5]. The additional concern is the increasing level of AMR, specifically extended-spectrum β-lactamase (ESBL) resistance, reported from *E. coli* that are recovered from fresh vegetables at the farm and retail levels [4,6]. ESBL-producing *E. coli* are mainly resistant to penicillins, cephalosporins, and aztreonam due to some variants of CTX-M, SHV, and TEM β-lactamases, among others, encoded by the genes *bla*_CTX-M_, *bla*_SHV_, and *bla*_TEM_, respectively [7]. Among the different ESBL types, CTX-M enzymes have emerged as the most widespread ESBL in animals and humans [6]. The risk of contaminated fresh salad products with pathogenic and antimicrobial resistant *E. coli* was highlighted throughout the Enterohaemorrhagic *E. coli* outbreak in Germany in 2011, caused by consuming fenugreek contaminated with CTX-M-15-producing *E. coli* O104:H4 clone, with over 4000 people becoming infected and more than 50 deaths reported [8].

In the United Arab Emirates, there is a dearth of research on the hygienic conditions of retail vegetables, particularly with regard to the microbial loads present on fresh salad products that are widely consumed [9]. Over the past few years, fresh salad vegetables in the UAE have been increasingly sourced from local soil-less farms that utilize vertical farms, hydroponic-based cultivation, and drip irrigation techniques to enhance water efficiency [10]. These farming systems are referred to as “smart farming”, which started adding value to the fresh produce market in the UAE, a country with scarce water resources and a harsh arid climate [11]. The presence of common bacterial groups, including *E. coli*, which is an essential hygienic indicator, may differ depending on the production system utilized (i.e., traditional versus smart agriculture) and geographic variations in the supply chain [12]. The contamination of fresh salad vegetables with *E. coli* and the subsequent spread of AMR within such products is of significant concern. *E. coli* is a prevalent Gram-negative bacterium that is responsible for both intestinal and extra-intestinal infections in humans [2]. However, there is a knowledge gap in the UAE regarding the extent of contamination and the AMR profile of *E. coli* in locally sourced and imported fresh salad vegetable products.

The objective of this study was to assess the presence of *E. coli* in retail fresh salad vegetables and to analyze the variability in contamination levels with respect to the production and origin of the products. Furthermore, we investigated the patterns of AMR and multidrug-resistance phenotypes in *E. coli* strains isolated from salad vegetables sold in the UAE. A subset of *E. coli* strains displaying multidrug resistance (MDR) was selected for whole-genome sequencing to gain insight into their resistance determinants, the prevalence of β-lactamase resistance genes, and other molecular typing characteristics. Overall, the findings of this study provide valuable insight into the spread of antimicrobial resistance in fresh salad vegetable produce, both locally and globally.

## 2. Material and Methods

### 2.1. Study Setting and Sample Collection

About 88% of the population inhabiting the seven emirates of the UAE is urban. The Emirate of Dubai is the most populous city in the UAE, while Abu Dhabi is the capital city of the UAE and the largest Emirate of the country; the vast majority of local agriculture takes place here. The total population residing in the emirates of Dubai and Abu Dhabi constitute about 65% of the country’s population [13]. Samples of fresh salad vegetables were purchased at the retail level to consider all possible contamination routes (including post-pack house transportation and handling), excluding those related to consumer food handling in the home. Supermarkets and vegetable markets in the Emirates of Dubai and Abu Dhabi were included in the sampling frame to represent the produce buying habits of a broad cross-section of consumers in the UAE urban settings and to enable the capture of sufficient samples from both domestic versus imported products and conventional versus smart farming cultivates; the latter farming system has emerged rapidly in the UAE over the past few years. The term smart farming is commonly used in the context of the UAE agriculture sector to refer to indoor soil-less farming where crops are grown in vertically stacked layers or shelves, using artificial lighting, climate control, and nutrient solutions. The shelves are equipped with grow lights and a hydroponic or aeroponic system to provide the necessary nutrients to the crops [10].

The sample size was calculated assuming a prevalence rate of 50%, a confidence level of 95%, and a margin of error of 5% [14]. As a result, a total of 400 samples were collected and tested over ten months, from May 2022 to January 2023 (Table 1). At the time of collection, the samples were inspected for freshness and the absence of soil, dirt, and visual signs of spoilage. Additionally, information on labeling and sample origin was recorded for all items. The samples were transported in a cold box at around 4 °C, securely sealed with sterile plastic wrap, and subjected to microbiological analysis within 24 h of collection.

### 2.2. Enumeration and Confirmation of E. coli

A portion of twenty-five grams of each sample was weighed into sterile homogenization bags and mixed with 225 mL Saline Peptone solution (NaCl 8.5 g L^−1^, Peptone 1.0 g L^−1^) in a BagMixer (Type 400; InterScience, Saint-Nom-la-Bretèche, France). Decimal dilutions (up to 10^−4^) were prepared with the same diluent, and 0.1 mL of each was used as inoculum for spread surface plating on Tryptone Bile Glucuronide Agar and followed by incubation at 44 °C for 24 h [15]. Up to three colonies, where β-glucuronidase-positive growth was indicated by blue or blue-green colonies, were examined for *E. coli* confirmation using PCR targeting for amplification of the *uidA* gene [16]. One confirmed colony per sample was subcultured for purification, and the isolate’s culture was stored in Brain Heart Infusion Broth (Oxoid, Basingstoke, UK) supplemented with 15% glycerol (HiMedia, Mumbai, India) at −80 °C.

### 2.3. Antibiotic Susceptibility Testing

Out of the fresh salad vegetable samples that tested positive for *E. coli*, a total of 145 isolates from various product types and origins were selected for analysis using the Kirby–Bauer method. The antimicrobial susceptibility phenotypes were assessed based on the Clinical Breakpoint values established by the Clinical and Laboratory Standards Institute (CLSI) [17]. The CLSI guidelines for human medicine were utilized in the analysis to align with the public health focus of the study. Antibiotics, such as ciprofloxacin (CIP [5 μg]), gentamicin (CN [10 μg]), ampicillin (AMP [10 μg]), azithromycin (AZM [15 μg]), cefoxitin (FOX [30 μg]), imipenem (IPM [10 μg]), and cefepime (FEP [30 μg]), were evaluated using the disc-diffusion method. The *E. coli* ATCC 25922 strain was included as quality control for each run. Isolates were categorized as MDR if they were non-susceptible to at least one agent in ≥three antimicrobial classes using CLSI breakpoints [18].

### 2.4. Whole-Genome Sequencing-Based Characterization

A commercial sequencing facility, Novogene, conducted Whole-Genome Sequencing (WGS) of 18 *E. coli* strains that were selected for being multidrug-resistant based on the Illumina NovaSeq platform PE150 (Illumina, San Diego, CA, USA). The assembled sequences were then uploaded to PathogenWatch (https://pathogen.watch) to verify species and serotypes (accessed on 7 March 2023). Multilocus sequence typing was performed in silico using the *E. coli* Achtman scheme obtained from PubMLST (https://pubmlst.org/bigsdb?db=pubmlst_ecoli_achtman_seqdef; accessed on 11 March 2023). The sequence types (STs) were calculated through Enterobase (accessed on 11 March 2023). To detect acquired resistance genes, ResFinder 4.1 was used using default parameters [19]. Plasmid replicon types were determined using PlasmidFinder version 2.1 with an identity percentage greater than 95% and a coverage cutoff higher than 90% [20]. The presence of the virulence genes was assessed using the VirulenceFinder version 2 using the default parameters [21]. SerotypeFinder 2.0 was used to predict the serotype, while CHTyper-1.0 was launched to detect the fumarase enzyme-coding house-keeping gene *fumC* and the type 1 fimbriae-specific adhesion-coding gene *fimH*, which were used to further categorize the isolates. The whole-genome fastq files of the paired-end sequence reads of the study isolates are available from the National Center for Biotechnology Information (NCBI) under the BioProject accession number PRJNA973558, with Sequence Read Archive (SRA) identifier SRP438328.

### 2.5. Data Analysis

The data on *E. coli* enumeration was recorded as the number of colony-forming units (CFU) per gram of fresh salad vegetable products. Results of the microbiological analysis were assessed against the national standard (UAE standard No: 1016/2002) [22] that described microbiological criteria for food stuffs, which aligns with internationally available criteria for *E. coli* examination of ready-to-eat foods (e.g., Health Protection Agency (HPA) in the UK [23]), where the unsatisfactory level of *E. coli* is indicated as ≥100 CFU/g.

To provide a descriptive summary of enumeration results, the counts were transformed to a logarithmic scale (base 10) to approximate a normal distribution. To investigate the relationship between selected variables and *E. coli* counts, the actual counts were analyzed using the Poisson regression model in the STATA statistical software, version 16.1 (STATA Corporation, Texas, TX, USA). However, the enumeration data were found to have a skewed distribution, and Poisson regression was not always the optimal model. Thus, a negative binomial model was employed to account for extra-Poisson variation [24]. Statistical significance was defined as differences with *p* values less than 0.05.

## 3. Results

### 3.1. Presence and Determinants of E. coli in Fresh Salad Vegetables

**Figure 1:**
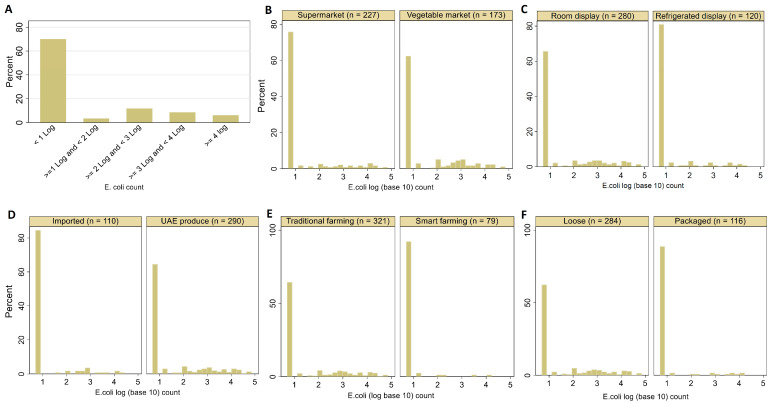
Variation in *E. coli* counts on fresh salad vegetables (*n* = 400) sampled from the United Arab Emirates retail concerning sampling site (**A**), sample display status (**B**), sample packaging status (**C**), the origin of samples (**D**), farming system (**E**), and the overall frequency distribution of *E. coli* counts in all samples (**F**), The bar below 1 log refer to samples below the limit of quantification.

As presented in Table 1, we sampled 11 types of fresh salad vegetable items (*n* = 400) from different retailers in Abu Dhabi (71.75%) and Dubai (28.25%). Using the direct plating method, *E. coli* was recovered from 30% (120/400) of the tested samples, of which arugula and spinach had a higher rate of *E. coli* recovery than other items (Table 1). The result presented in Figure 1A shows that 70% (280/400) of the samples were below the quantification limit of *E. coli* (10 CFU/g). On the other hand, 26.5% (106/400) of the samples had a count of ≥100 CFU/g and were thus categorized as having an unsatisfactory *E. coli* level (Table 1 and Figure 1A).

Figure 1 describes the variation in *E. coli* counts distribution concerning different variables. The samples in this study were collected from two sites, supermarkets (56.75% (227/400)) and vegetable markets (43.25% (173/400)). The majority of the samples were displayed at room temperature (not refrigerated, 70% (280/400)) and presented as loose (not packaged, 71% (284/400)). Fresh salad vegetable items from local producers were more represented (72.50% (290/400)), and 19.75% (75/400) of the samples were produced using local smart farming.

As presented in Table 2, the sampling site, sample display, and sample packaging status had no significant effect on *E. coli* counts in fresh salad vegetables. On the other hand, the model indicated that samples from local produce had a significantly higher (*p* =< 0.001; Coefficient = 1.861) *E. coli* count than imported samples. The analysis also indicated that fresh salad vegetables from the smart farming system had significantly fewer (*p* =< 0.001; Coefficient = −2.818) *E. coli* than those from traditional produce farming (Table 2).

### 3.2. Antimicrobial Resistance in E. coli from Fresh Salad Vegetables

From fresh salad vegetable samples with countable *E. coli* results (*n* = 120), one or more isolates (if varied in colony morphology), were selected for screening their resistance against 12 agents representing 11 antimicrobial categories (Table 3); thus, a total of 145 isolates were screened. The isolates exhibited the highest phenotypic resistance toward ampicillin (20.68% (30/145)), followed by tetracycline (20% (29/145)), and then against trimethoprim-sulfamethoxazole (10.35% (15/145)). None of the isolates were resistant to imipenem, and a limited proportion (<10%) of the isolates showed resistance to clinically significant antibiotics such as ceftriaxone, cefotaxime, ciprofloxacin, and azithromycin (Table 3). A total of 20 (13.79%) of the 145 *E. coli* isolates characterized from fresh salad vegetable samples exhibited a multidrug-resistant phenotype (Table 4).

### 3.3. Genomic Characterization of Multidrug-Resistant E. coli Isolates

A total of 18 out of the 20 multidrug-resistant *E. coli* isolates were further characterized using whole-genome sequencing. As indicated in Table 5, 12 sequence types (STs) were detected among the 18 sequenced MDR *E. coli* isolates. The most frequently sequence types were ST58 and ST7588, with each identified in three unrelated isolates. Of the 15 isolates, 1 or more plasmid incompatibility types were featured, with IncFIB being the most represented in 7 of them. Only one CTX-M β-lactamases gene was presented, and that was *bla*_CTX-M-15_, which was prevalent in 50% (9/18) of the *E. coli* isolates characterized from fresh salad vegetable samples. Out of the 18 isolates, 6 harbored *bla*_CTX-M-15_ and *bla*_TEM-1B_ genes concurrently (Table 5). The most common resistance genes for aminoglycosides were *aph(3″)-lb* and aph*(6)-ld*, with both genes concurrently presented in nine isolates—only one isolate (207.1) harbored *mph(A)* gene which conferred resistance to the macrolide (Table 5). The tetracycline resistance genes *tet(A)* and, to a lesser extent *tet(B)* were present in 17 of the 18 *E. coli* isolates (Table 5). As presented in Table 5, various serotypes were identified based on WGS analysis. One isolate (194.2) harbored serotype O45, one of the six non-O157 serovars most commonly identified as causing foodborne illness. Most isolates (11/18) were related to phylogroup B1 (Table 5).

Based on WGS analysis, the results in Table 6 indicate the presence of 16 virulence genes that were associated with extra-intestinal and intestinal *E. coli* pathotypes. Among the characterized multidrug resistant isolates, the following genes were the most prevalent: Curli fimbriae subunit protein (*csgA*, 17/18), Hemolysin E (*hlyE*, 12/18), Type 1 fimbrial adhesin (*fimH*, 11/18), and Increased serum survival protein (*iss*, 9/18) (Table 6).

## 4. Discussion

The consumption of fresh salad vegetables, both leafy and non-leafy, in their raw form, exposes individuals to foodborne bacteria, some of which may be antibiotic-resistant [2,4]. Our study presents the results of the first investigation of *E. coli* contamination levels and patterns of antimicrobial resistance in fresh salad vegetables in the UAE. There are numerous potential origins for the contamination of *E. coli* in raw salad vegetables, including soil, water, and the environment [25]. The presence of *E. coli* in food suggests recent exposure to fecal matter, either directly or indirectly, and indicates a likelihood that other enteric pathogens known to cause foodborne gastroenteritis and bacterial diarrhea are present [2].

Our findings demonstrate that a substantial proportion (26.5%) of fresh salad vegetables, notably arugula and spinach, sampled from retail outlets in the UAE contain an unsatisfactory level of *E. coli* counts (>100 CFU/g). This result is consistent with previous research indicating that leafy vegetables, such as arugula and spinach, have a greater propensity for surface attachment, which increases the chances of survival of certain bacterial groups [4,12]. When using the same interpretation criteria for an unsatisfactory level, studies conducted in industrialized nations reported lower rates of unsatisfactory *E. coli* counts, such as 1.5% in the UK [25], 4% in Portugal [26], and 0% in Australia [27]. Conversely, studies conducted in other countries have reported higher rates of *E. coli* contamination in fresh salad vegetables compared to our survey in the UAE, such as 100% detection in coriander leaves in Bangladesh [28] and 34% contamination in leafy and non-leafy vegetables in Pakistan [29]. However, caution should be exercised when directly comparing our results with those of other studies due to differences in methodology, geography, climate, and production practices between study settings. As the first of its kind in the UAE, this study will serve as a benchmark for future efforts to monitor the hygienic quality of fresh salad vegetables intended for consumption by consumers.

Aside from assessing the level of *E. coli* counts, we also aimed to investigate the association between certain determinant variables and the enumeration outcomes of *E. coli* in fresh salad vegetables. Our study data indicated that samples sourced from local (UAE-based) produce had higher *E. coli* counts compared to imported fresh salad vegetables. Similar to our findings, a study conducted in New Zealand revealed that all imported fresh produce samples had a satisfactory *E. coli* count, whereas at least 54% of samples with marginal and unsatisfactory *E. coli* counts were attributed to domestically grown leafy greens [30]. Exporting fresh produce establishments typically undergo rigorous certification and control, including microbial quality checks, at both exporting and importing countries’ ends. Additionally, imported vegetables analyzed in our study were typically packaged, preventing direct contact by workers and consumers. Moreover, imported fresh produce is usually shipped under controlled cold chain conditions, which might contribute to its overall microbial quality and safety [31].

On the other hand, our study’s statistical analysis indicated that fresh salad vegetables sourced from smart-farmed samples had significantly lower *E. coli* counts than those from conventional production systems; all of the samples tested from smart-farms met the designated national microbiological criteria for *E. coli*. Unlike conventional farming, smart farming, which uses soil-less indoor cultivation methods, such as hydroponics and aeroponics, provides a more controlled environment that is easier to manage and prevents microbial contamination in the cultivation facility [32]. Several studies have demonstrated lower contamination levels and the absence of generic *E. coli* in soil-less-grown crops compared to soil-grown crops [33,34]. Nevertheless, a *Salmonella typhimurium* outbreak has been linked to leafy greens from a hydroponic farm in the USA. Without good farming and agricultural practices, hydroponics does not guarantee plant health or fresh produce’s microbial safety. All components of smart farming systems, including hydroponic facilities, should be regularly monitored for indicator bacteria and foodborne pathogens to assess the potential risk of crop contamination [33].

Antimicrobial resistance surveillance programs mainly concentrate on animal-derived food products; however, it is equally important to monitor antimicrobial resistance in non-animal-derived food. The present study focused on *E. coli* isolates found in fresh salad vegetables in the UAE. These isolates exhibited the highest phenotypic antimicrobial resistance towards ampicillin, tetracycline, and trimethoprim-sulfamethoxazole. The use of antibiotics in animal feed and as veterinary drugs is increasing, which poses a threat to the entire agriculture ecosystem, including raw vegetable production systems, soil, and groundwater quality [35]. Our findings suggest that fresh produce vegetables could also serve as a source for multidrug-resistant *E. coli*. It is noteworthy that all MDR isolates (*n* = 20) identified in our study were sourced from locally produced leafy salad vegetable items. Along with potential preharvest contamination sources, poor personal hygiene by vendors and agricultural workers, poor sanitation facilities, and unhygienic conditions at marketplaces are also linked to the contamination of fresh vegetables and fruits with antimicrobial-resistant bacteria [36]. The prevalence of antibiotic resistance in *E. coli* is a significant concern since it is the most common Gram-negative bacterial pathogen that causes both intestinal and extra-intestinal infections in humans [2].

In this study, whole-genome sequencing (WGS) was utilized to characterize the MDR *E. coli* strains recovered from fresh salad vegetables sold in the UAE. The analysis of these isolates revealed several features that pose a potential risk to human health. Notably, the two most frequently identified STs were clinically relevant sequence types, including *E. coli* ST58 and ST7588. ST58 has recently emerged as a globally disseminated uropathogenic clone that can lead to sepsis [37]. Our findings show that all (three) *E. coli* ST58 isolates identified in our study belong to the environmental/commensal phylogroup B1, in contrast to most pandemic extra-intestinal pathogenic *E. coli* (ExPEC) that belong to pathogenic phylogroup B2 [38]. ST58 has been found in healthy and diseased food-producing animals, poultry farm-associated flies, manure, and water [37], indicating an environmental contamination source for its introduction to fresh salad vegetables. ST7588 was also frequently detected among MDR *E. coli* in this study. One ST7588 isolates (arugula, isolate 194.2) was identified as the O45 serotype, one of the primary (top six non-O157:H7) Shiga toxin-producing serotypes of *E. coli* that has been identified as a cause of sporadic cases of bloody diarrhea in humans [39]. The prevalence of potentially pathogenic *E. coli* in leafy salad vegetable items highlights the need for cost-effective, accurate, and rapid identification systems to decrease the public’s exposure to *E. coli* infection.

Moreover, the study revealed a potential high endemicity of the international high-risk ESBLs gene type CTX-M-15 in *E. coli* from fresh leafy salad vegetables. CTX-M-15 was the only CTX-M type identified and was present in 50% of the isolates characterized in this study. CTX-M-15 is known to be the most clinically relevant ESBL worldwide [6]. The contamination of fresh leafy vegetables by critical priority pathogens is of great concern since these foods are consumed raw, increasing the risk of human exposure to ESBL producers and other antibiotic-resistant bacteria of clinical significance [2]. Although ingesting ESBL-producing bacteria may not have an immediate clinical health implication, colonization by this pathway may contribute to the transfer of antibiotic-resistance genes to other bacterial species in the gut microbiota. Consequently, a potential threat to human health may be associated with future endogenous infections, especially in immunosuppressed patients, where therapeutic failure can occur [40]. It is crucial to closely monitor the extent of CTX-M-15 in fresh leafy vegetables from farm to retail and investigate the factors that might contribute to its introduction and spread in *E. coli* inhabiting the local production environment.

This study’s virulence factors analysis of MDR *E. coli* isolated from fresh salad vegetables points to the abundance of several extra-intestinal and intestinal pathotypes associated genes. The ability of a microorganism to cause diseases depends not only on its virulence factors but also the host’s underlying determinants [41]. The gene *csgA* was the most frequently identified; it has been indicated as an important virulence factor enabling *E. coli* to effectively colonize intestinal epithelium, especially in individuals with inflammatory intestinal disorders. Moreover, *csgA* gene has been widely distributed in uropathogenic *Escherichia coli* (UPEC), being involved in adhesion to human bladder cell surfaces and biofilm development [42,43]. Harboring many bacterial virulence factors was reported to affect the severity and the extent of infection of any pathogenic microorganisms [41]. The abundance of virulent factors among MDR *E. coli* calls for a One Health antibiotic stewardship program and harmonized screening of the spread of antimicrobial resistance in leafy salad vegetables. WGS analysis of *E. coli* populations in fresh salad vegetables is a powerful tool that characterizes bacterial typing, antimicrobial resistance, and virulence profiling in a relatively cost-efficient workflow.

## 5. Conclusions

This study presents a novel evaluation of the level of *E. coli* contamination and the prevalence of antimicrobial resistance in fresh salad vegetables sold in the UAE. The study was limited to sampling from the two most populated Emirates, Dubai and Abu Dhabi, and future studies are recommended to be more inclusive. As the study was concerned with sampling leafy greens, some important salad vegetable items were not included (e.g., cucumber and tomato). Despite these limitations, the findings of this study address a gap in the existing literature on the hygienic quality and safety of plant-based foods in the Middle East. While approximately 75% of the tested samples showed acceptable levels of *E. coli*, a considerable proportion of samples had an unsatisfactory bacterial load of ≥100 CFU/g. These outcomes indicate the need for continuous monitoring and improvement of pre-harvest and post-harvest conditions to enhance the hygienic quality of fresh produce in the UAE. Encouragingly, samples obtained from smart farming sources showed significantly lower *E. coli* counts, a positive finding given the increasing popularity of soil-less farming techniques, such as hydroponic and aeroponic systems, in the UAE. Although only a small number of *E. coli* isolates in this study were identified as MDR, they carried clinically significant antimicrobial resistance genes, such as CTX-M-15, and virulence genes related to uropathogenic and intestinal pathotypes, and some globally recognized sequence types previously associated with human illness. In summary, our findings suggest that fresh salad vegetables marketed for human consumption may be an undercover vehicle for spreading international clones of critical priority resistance genes, such as ESBLs, to humans. Overall, our study highlights the importance of continued research and monitoring of antimicrobial resistance at the human–food interface. Fresh produce and leafy greens are an important part of our healthy diets, but consumers should aware of the importance of exercising hygienic eating and handling practices when consuming fresh vegetables to minimize any potential health risks.

## Figures and Tables

**Table 1:** Overview of *E. coli* contamination for different fresh salad vegetable samples (*n* = 400) collected from retail sources in the United Arab Emirates.

Product Item	Total Samples No. (%)	No. of Samples Positive for *E. coli* (%)	No. of Samples with Unsatisfactory *E. coli* Counts (%) **
Arugula	51 (12.75)	40 (78.43)	36 (70.59)
Cabbage	36 (9.00)	— ***	—
Coriander	31 (7.75)	9 (29.03)	7 (22.58)
Dill	18 (4.50)	10 (55.56)	9 (50.00)
Iceberg lettuce	31 (7.75)	—	—
Romaine lettuce	51 (12.75)	5 (9.89)	5 (9.80)
Lettuce (others) *	48 (12.00)	2 (4.17)	2 (4.17)
Onion leaves	15 (3.75)	1 (6.67)	1 (6.67)
Parsley	39 (9.75)	15 (38.46)	13 (33.33)
Spinach	49 (12.25)	32 (65.31)	30 (61.22)
Mixed packs	31 (7.75)	6 (19.35)	3 (9.68)
Total	400 (100.00)	120 (30.00)	106 (26.50)

* Other types of lettuce varieties include Lollo Rosso, Armela, Boston green, Little Gem, Garden radish pink, and Butterhead. ** ≥100 CFU/g. *** Not detected.

**Table 2:** Negative binomial regression analysis results on the association between selected determinant variables and *E. coli* counts on fresh salad vegetables (*n* = 400) sampled from the United Arab Emirates retail.

Determinant Variable	Coefficient	Standard Error	*p*-Value	95% Confidence Interval
Sampling site: Vegetable market vs. supermarket	0.081	0.311	0.792	−0.528; 0.691
Sample display status: Refrigerated display vs. room temperature display	0.526	0.790	0.505	−1.021; 2.075
Sample packaging status: Packaged vs. loose	−0.241	0.830	0.771	−1.869; 1.386
The origin of samples: Local vs. imported	1.861	0.327	<0.001	1.218; 2.504
Farming system: Smart farming vs. traditional farming	−2.818	0.535	<0.001	−3.867; −1.768

**Table 3:** Antimicrobial resistance phenotypes of *E. coli* isolates (*n* = 145) recovered from fresh salad vegetable products sampled from retail at the United Arab Emirates.

Antimicrobial Category	Antimicrobial Agent	No. of *E. coli* Isolates (*n* = 145)		
		Resistant *n* (%)	Intermediate *n* (%)	Susceptible *n* (%)
Fluoroquinolone	Ciprofloxacin (CIP)	9 (6.20)	80 (55.18)	56 (38.62)
Phenicol	Chloramphenicol (C)	10 (6.89)	1 (0.68)	134 (92.42)
Aminoglycosides	Gentamicin (CN)	4 (2.75)	2 (1.37)	139 (95.86)
Tetracyclines	Tetracycline (TET)	29 (20.00)	9 (6.20)	107 (73.78)
2nd generation Cephalosporins	Cefoxitin (FOX)	1 (0.68)	2 (1.37)	142 (97.94)
3rd generation Cephalosporins	Cefotaxime (CTX)	10 (6.89)	2 (1.37)	133 (91.72)
	Ceftriaxone (CRO)	11(7.58)	2 (1.37)	132 (91.04)
4th generation Cephalosporins	Cefepime (FEP)	1 (0.68)	9 (6.20)	135 (93.11)
Carbapenems	Imipenem (IPM)	0 (0.00)	2 (1.38)	143 (98.62)
Sulfonamides	Trimethoprim-sulfamethoxazole (SXT)	15 (10.35)	1 (0.68)	129 (88.96)
Penicillin	Ampicillin (AMP)	30 (20.68)	2 (1.37)	113 (77.94)
Macrolides	Azithromycin (AZM)	8 (5.51)	1 (0.68)	136 (93.80)

**Table 4:** Multidrug resistance (per antimicrobial classes) phenotypes found in *E. coli* isolates (*n* = 145) recovered from fresh salad vegetable products sampled from retail sources in the United Arab Emirates.

No. Antimicrobial Classes	No. of Isolates (%)	Co-Resistance Patterns * (No. of Isolates per Pattern)	Codes of Isolates Characterized by Whole-Genome Sequencing
Three	7 (35)	3rdGC-T-P (3)	**41.1, 43.2, 153.1**
		T-S-P (1)	**55.1**
		Ph-3rdGC-P (1)	**56.1**
		3rdGC-P-4thGC (1)	**178.1**
		T-P-M (1)	**207.1**
Four	6 (30)	3rdGC-T-S-P (3)	**34.1, 194.2, 350.1**
		FQ-A-T-P (1)	**11.1**
		FQ-T-S-P (1)	**77.2**
		Ph-T-S-P (1)	**242.1**
Five	7 (35)	FQ-Ph-T-S-P (3)	**77.1**, 124, **266.2**
		A-2ndGC-S-P-M (1)	87.1
		T-3rdGC-S-P-M (1)	**345.2**
		A-T-3rdGC-S-P (1)	**346.2**
		FQ-Ph-A-T-P (1)	**359.1**
Total	20 (100.00)		18/20 **

* Fluoroquinolone, FQ; Phenicol, Ph; Aminoglycosides, A; Tetracyclines, T; 2nd generation Cephalosporins, 2ndGC; 3rd generation Cephalosporins, 3rdGC; 4th generation Cephalosporins, 4thGC; Sulfonamides, S; penicillins, P; Macrolides, M. ** A total of 18 (marked in bold) out of the 20 identified multidrug-resistant *E. coli* were further characterized using whole-genome sequencing (isolated.

**Table 5:** Whole-genome sequencing inferred characterization of multilocus sequence types (ST), plasmid incompatibility types, antimicrobial resistance genes, and predicted serotype and phylogroups of 18 multidrug-resistant *E. coli* isolated from salad vegetable products sampled from the United Arab Emirates retail.

Isolate	Product Item *	ST	Plasmid Incompatibility Types	Antimicrobial Resistance Genes				
				Beta-Lactam	Quinolone	Macrolide	Aminoglycoside	Lincosamide
**11.1**	Parsley	1642	IncFIB, IncFIA, IncY	*blaTEM-1B*	*x*	*x*	*aph(3″)-lb, aph(6)-ld, aph(3′)-la, aac(3)-lld, aadA17*	*lnu(F)*
34.1	Rocket	58	IncFIB	*blaCTX-M-15, blaTEM-1B*	*qnrS1*	*x*	*aph(3″)-lb, aph(6)-ld*	*x*
41.1	Arugula	58	IncFIB	*blaCTX-M-15, blaTEM-1B*	*qnrS1*	*x*	*aph(3″)-lb, aph(6)-ld*	*x*
43.2	Arugula	1727	IncFIB	*blaCTX-M-15, blaTEM-1B*	*qnrS1*	*x*	*aph(3″)-lb, aph(6)-ld*	*x*
55.1	Parsley	1294	IncFIB	*blaTEM-1B*	*qnrS1*	*x*	*aph(3″)-lb, aph(6)-ld*	*x*
56.1	Coriander	1727	IncY	*blaCTX-M-15*	*qnrS1*	*x*	*aph(3″)-lb, aph(6)-ld*	*x*
77.1	Parsley	206	IncFII, IncX4, IncR	*x*	*x*	*x*	*aadA24, aph(3′)-la, aadA2*	*lnu(G)*
77.2	Parsley	206	IncFII, IncX4, IncR	*x*	*x*	*x*	*aph(3′)-la, aadA1*	*lnu(G)*
153.1	Arugula	155	x	*blaCTX-M-15*	*qnrS1*	*x*	*x*	*x*
178.1	Parsley	2161	x	*blaCTX-M-15*	*qnrS1*	*x*	*x*	*x*
194.2	Arugula	7588	IncFIB	*blaCTX-M-15, blaTEM-1B*	*qnrS1*	*x*	*aph(3″)-lb, aph(6)-ld*	*x*
207.1	Spinach	10	IncN	*blaTEM-1B*	*qnrS1*	*mph(A)*	*aadA17*	*lnu(F)*
242.1	Lettuce	58	IncR	*blaCARB-2*	*qnrS1*	*x*	*aadA1, aadA2b*	*x*
266.2	Coriander	2206	x	*blaTEM-1B*	*x*	*x*	*aph(3″)-lb, aph(6)-ld, aadA1*	*x*
345.2	Arugula	7588	IncR, IncFIB	*blaCTX-M-15, blaTEM-1B*	*qnrS1*	*x*	*aph(3″)-lb, aph(6)-ld*	*x*
346.2	Arugula	328	IncFII	*blaTEM-1B*	*x*	*x*	*aph(3″)-lb, aph(6)-ld*	*x*
350.1	Arugula	7588	IncFIB	*blaCTX-M-15, blaTEM-1B*	*qnrS1*	*x*	*aph(3″)-lb, aph(6)-ld*	*x*
359.1	Parsley	224	IncHI2/	*blaTEM-1A*	*x*	*x*	*aph(3″)-lb, aph(3′)-la, aph(6)-ld, aadA1, aac(3)-lla*	*x*
			IncHI2A					

* All products from which the 18 whole-genome sequenced isolates were identified as locally grown (UAE-based) fresh salad vegetable samples.

**Table 6:** The common extra-intestinal and intestinal virulence-related genes identified based on whole-genome sequencing analysis of 18 multidrug-resistant *E. coli* isolated from salad vegetable products sampled from retail sources in the United Arab Emirates.

Gene	Frequency	Gene Function/Description	ExPEC Pathotype *	Intestinal Pathotype *
*csgA*	17	Curli fimbriae subunit protein	UPEC, SEPEC, APEC	EPEC, EHEC
*hlyE*	12	Hemolysin E		EAEC
*fimH*	11	Type 1 fimbrial adhesin fimh	UPEC, NMEC, SEPEC, APEC	
*iss*	9	Increased serum survival protein	NMEC, SEPEC, APEC	
*afaA*	2	Afimbrial adhesin A	UPEC	
*iucC*	1	Aerobactin synthase component C	UPEC, APEC	EIEC
*espA*	1	Type III secretion system protein espa		EPEC, ETEC
*espF*	1	Type III secretion system protein espf		EPEC, EHEC
*espB*	1	Type III secretion system protein espb		EPEC
*nleA*	1	Non-LEE-encoded effector A		EPEC
*espJ*	1	Type III secretion system protein espj		EPEC, EHEC
*tir*	1	Translocated intimin receptor		EPEC, EHEC
*iutA*	1	Aerobactin receptor		EIEC
*afaD*	1	Afimbrial adhesin D	UPEC	
*traT*	1	Serum resistance-associated protein trat	NMEC, SEPEC, APEC	
*irp2*	1	Iron-regulated protein 2	NMEC	

* Extra-intestinal (ExPEC) and intestinal pathogenic *E. coli* (IPEC)—abbreviations: enteropathogenic *E. coli* (EPEC), enterohemorrhagic *E. coli* (EHEC), enterotoxigenic *E. coli* (ETEC), enteroaggregative *E. coli* (EAEC), diffusely adherent *E. coli* (DAEC), enteroinvasive *E. coli* (EIEC), extra-intestinal pathogenic *E. coli* (ExPEC), uropathogenic *E. coli* (UPEC), neonatal meningitis *E. coli* (NMEC), sepsis-associated *E. coli* (SEPEC), avian pathogenic *E. coli* (APEC), and mammary pathogenic *E. coli* (MPEC).

## Data Availability

The data presented in this study is available in the article. Whole-genome sequence data are available from the National Center for Biotechnology Information (NCBI) under the BioProject accession number PRJNA973558; SRA Experiment identifier (SRP438328).
